# Understanding antibody–target antigen interactions and the avidity effect using mathematical modelling

**DOI:** 10.1098/rsif.2024.0710

**Published:** 2025-07-09

**Authors:** Luke Heirene, Helen Byrne, Armin Sepp, Eamonn Gaffney, James Yates

**Affiliations:** ^1^ Mathematical Institute, University of Oxford, Oxford, UK; ^2^ Certara UK Limited, Sheffield, UK; ^3^ GSK plc, Stevenage, UK

**Keywords:** antibodies, immunotherapies, avidity, mathematical model

## Abstract

Immunotherapies are designed to exploit the immune system to target pathologies such as cancer. Monoclonal antibodies (mAbs) are an important class of immunotherapies that induce anti-tumour effects. Fundamental to the success of mAbs in cancer treatments are their interactions with target antigens. For example, binding multiple antigens, increasing binding affinity, termed the avidity effect, has been shown to impact treatment outcomes. However, there has been limited theoretical analysis addressing the impacts of antibody–antigen interactions on avidity, potency and efficacy. Hence, our aim is to use a mathematical model to develop insight on these impacts. We analyse an ordinary differential equation model of bivalent, monospecific IgG antibodies binding to membrane antigens and show that the ratio of antibody to antigen number impacts quantities that contribute to mAb potency and efficacy, such as antigen occupancy, and whether an antibody can bind both its antigen-binding arms. A global parameter sensitivity analysis shows that antigen occupancy and the ratio of bound antibody to total antigen number are sensitive to the antibody–antigen binding rates only for high antibody concentrations. We also identify parameter ranges in which the avidity effect is predicted to be large. These results could be used in the preclinical development of mAb therapies by predicting conditions that enhance mAb potency, efficacy and the avidity effect.

## 1. Introduction

The immune system can target infections and diseases such as cancer [1]. It comprises the innate system, which responds to a broad range of threats within the body, and the adaptive system, which is highly specific to its target [2,3]. The innate and adaptive systems work in unison to prevent the development of cancer and to destroy any cancer cells that arise [4].

However, tumour cells can disrupt the immune response in a variety of ways. For example, they can inhibit the immune response by expressing inhibitory immune checkpoint receptors such as the programmed cell death-ligand 1 (PD-L1) [5]. One aim of cancer immunotherapies is to mitigate the ability of tumour cells to disrupt the immune system while another is directly enabling the immune system to target the cancer.

Anti-tumour monoclonal antibodies (mAbs) are a class of immunotherapies that have had a major clinical impact via both mechanisms [6]. For example, mAbs can induce anti-tumour effects by binding to immune checkpoint receptors on tumour cells and, in so doing, inhibit the ability of tumour cells to suppress the immune response [7]. MAbs can also induce their anti-tumour effects through antibody effector functions, where they recruit and stimulate other parts of the immune system to target and kill cancer cells [8]. An important antibody effector function is antibody-dependent cellular cytotoxicity (ADCC), where mAbs that bind to cancer cells enable immune cells to recognize and then actively lyse the cancer cells [9].

Regardless of how mAbs induce their anti-tumour effects, central to their potency and efficacy are their interactions with target antigens, where potency is defined as the expression of drug activity for a given concentration or the amount of drug required to produce a defined effect, while efficacy is defined as the maximum response that can be achieved regardless of dose. Mazor *et al*. [10] highlight the relationship between antibody–antigen interactions and effector function potency and efficacy. By modifying the affinity of a variety of mAbs for their target antigen, Mazor *et al*. [10] showed that lower affinity variants exhibited improved effector function. Mazor *et al*. [10] hypothesized that lower affinity variants were more likely than higher affinity variants to form monovalent than bivalent bonds. Increased levels of monovalent binding increase the number of antibodies per target antigen that can activate an immune cell to kill a tumour cell and, thereby, increase both effector function potency and efficacy. Increased target antigen occupancy has also been found to enhance immune checkpoint inhibitors [11].

**Figure 1.**
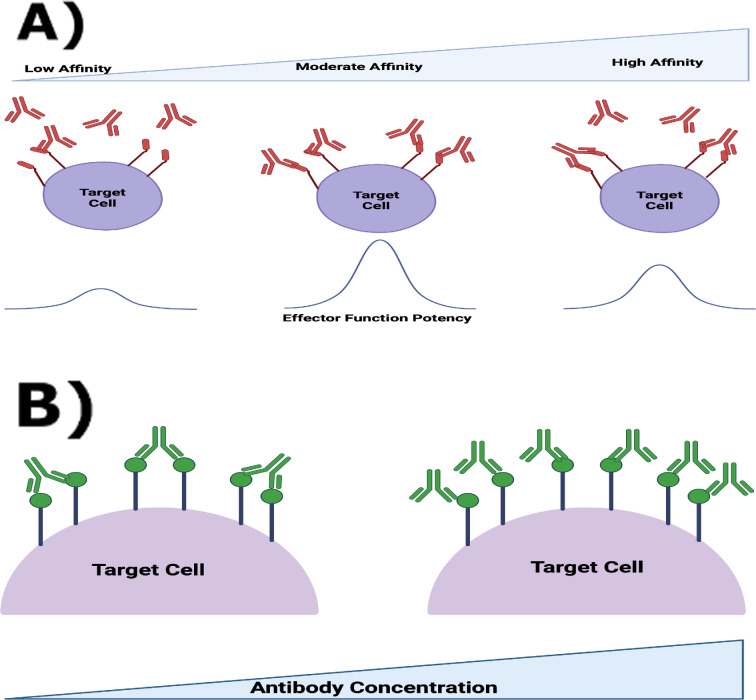
Schematic detailing the key antibody–antigen interactions involved in mAb therapies. (A) Enhancing effector function potency by modifying the antibody’s affinity for its target antigen (adapted from [10]). Effector function potency is improved for antibodies with moderate affinity due to the increased number of antibodies bound to the cell. The increased number of antibodies bound to the cell is due to increased levels of monovalent binding. Antibodies with high affinity are more likely to be bivalently bound and, therefore, the total number of antibodies bound to the cell is reduced. The curves depicted are a representation of the resulting potency from different affinity mAb variants. (B) The binding state of the antibody changes in terms of antibody concentration [12]. For low antibody concentrations, antibodies are primarily bivalently bound. As the antibody concentration increases sufficiently, antibodies are primarily monovalently bound. Created with Biorender.com.

In a separate experimental work, Bondza *et al*. [12] suggested that the proportion of monovalently and bivalently bound antibodies changes as antibody concentration varies. A mAb can engage cell surface targets both in monovalent and bivalent modes, depending on the target surface concentration, the mAb volume concentration, affinity and assuming unhindered target accessibility at both binding steps. At very low receptor numbers, binding can be predominantly monovalent as the complex dissociates before cross-linking, while at high mAb concentrations, the levels required for monovalent binding can be non-physiological. A schematic of the key antibody–target interactions detailed in [10] and [12] is presented in figure 1. Together, the works of Mazor *et al*. [10] and Bondza *et al.* [12] show how antibody–antigen interactions on the surface of the target cell can alter the antibody’s binding state and impact mAb potency and efficacy. However, there is no consensus about which of the parameters governing antibody–antigen interactions are most important for mAb potency and efficacy. For example, is it better to have a strong binding affinity or a larger number of target antigens? How does this change as the antibody concentration varies? As a first step towards answering these questions, in this article, we use mathematical modelling to investigate how factors that regulate antibody–antigen interactions (e.g. on- and off-binding rates, antibody concentration and antigen density) impact the potency and efficacy of mAb therapies, as quantified, for instance, by antigen occupancy and the number of bound antibodies.

When developing a mAb therapy, a key consideration is how the number of antigens an antibody can bind, termed its valency, affects its target binding dynamics. Consider, for example, a monospecific immunoglobulin G (IgG) antibody, a common type of bivalent antibody. After binding to a target antigen, the antibody can bind to a second target antigen with its remaining binding arm. Binding multiple antigens increases the experimentally measured binding affinity, termed the ‘avidity’ effect [13]. Some cells infected with pathogens, such as a virus, can evade an avidity effect if the surface proteins targeted by the antibodies are immobile and sparsely distributed across the cell membrane. In such cases, it is rare for an antibody to bind both of its arms, resulting in lower overall binding affinity and, hence, smaller levels of antibody binding at non-saturating antibody concentrations [14]. This is just one example where avidity, or lack thereof, is known to contribute to the antibody therapeutic effect. For other examples, see the review by Oostindie *et al*. [13].

Clearly, the avidity effect is an important feature of antibody–antigen interactions. However, there is no consensus about the conditions under which the avidity effect is large for mAb binding. Mathematical modelling can be used to investigate whether there are certain ranges of total target antigen densities and binding affinities that result in a large avidity effect.

Several authors have developed mathematical models that describe antibody binding to cells [15–18]. These models differ in the mAb–antigen interaction under consideration (e.g. monospecific or bispecific) and the way in which the second arm of the antibody binds to its target antigen. For example, Sengers *et al*. [18] assume that binding of the second arm is limited by surface diffusion of target antigens, whereas Rhoden *et al.* [17] assume that binding of the second arm is driven by antigen levels within reach of the bound arm of the antibody.

Here, we focus on monospecific, bivalent antibodies. We will extend the model of bivalent ligand–antigen binding given in Perelson & DeLisi [15] with two main aims. First, we aim to investigate how simultaneously varying system parameters, such as antigen density and binding affinity, impact quantities of interest such as the antibody binding state, the number of bound antibodies and antigen occupancy. Second, we aim to identify parameter ranges that induce a strong avidity effect.

## 2. Methods

In this section, we introduce a time-dependent mathematical model that describes the binding of a monospecific, bivalent antibody to target antigens on the cell membrane. We formulate our model as a system of ordinary differential equations (ODEs).

Our model is based on an existing model of bivalent ligand binding presented in Perelson & DeLisi [15]. Unlike Perelson & DeLisi [15], we do not assume that the ligand (antibody in this case) is always in excess. We formulate our model in units of the number of antibody or antigen rather than concentration in order to more clearly calculate quantities of interest like antigen occupancy and the ratio of bound antibody to antigen. As in Perelson & DeLisi [15], we neglect spatial effects by assuming that the system is well mixed and that target antigens are distributed uniformly over the cell membrane. The dependent variables are the number of unbound target antigens, r(t); the number of unbound antibodies, $A_{0}(t)$; the number of monovalently bound antibodies, $A_{1}(t)$; and the number of bivalently bound antibodies, $A_{2}(t)$.

**Figure 2.**
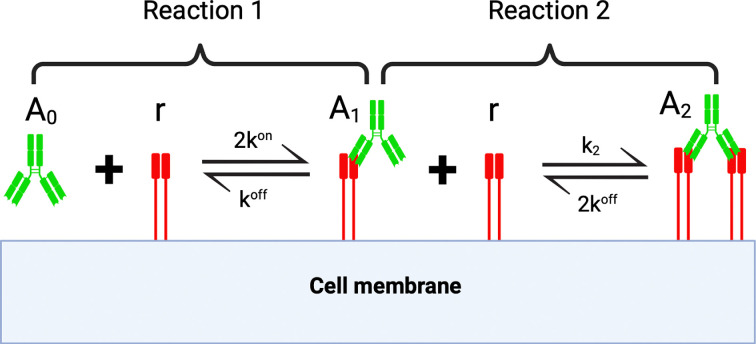
Reaction scheme for antibody binding on the surface of a target cell. The arm of an unbound antibody, $A_{0}$, binds reversibly with a free target antigen, r, at a rate $k^{\text{on}}$, to form a monovalently bound antibody $A_{1}$ and can dissociate at a rate $k^{\text{off}}$. $A_{1}$ can bind another free antigen at a rate $k_{2}$, to form a bivalently bound antibody $A_{2}$. A bivalently bound antibody can dissociate one of its bound arms away from the target antigen independently of, and at the same rate as, the monovalently bound arm. Note, in an abuse of notation, here $A_{0}$ denotes a single unbound antibody, whereas in equations (2.1)–(2.4), it denotes the total number of unbound antibodies (similarly for all other variables). Created with Biorender.com.

The mathematical model we consider describes how a monospecific, bivalent antibody binds to a target antigen on a single tumour cell as depicted in figure 2. We assume an unbound monospecific antibody binds reversibly with one of its arms to a free target antigen, to form a monovalently bound antibody–antigen complex. The monovalently bound antibody reversibly binds a second free antigen with its unbound arm, to form a bivalently bound antibody. At this stage, each antigen-bound arm may dissociate from its antigen. The rate at which one arm dissociates is assumed to be equal to, and independent of, the rate at which the other arm dissociates.

For simplicity, we neglect antigen internalization. This is justified because, typically, the time scale of antigen internalization is much slower than the time scale on which antibody binding takes place on the surface of a cell. For example, the internalization half-life of mavrilimumab is approximately 30 min to 2 h while the time scale of antibody binding can be much less than a second for high antibody concentrations [19,20]. However, we acknowledge that this may not be a reasonable assumption for some ligands that have a fast internalization rate, such as the transferrin receptor [21].

Under the above assumptions, our mathematical model can be written as follows:


(2.1)drdt=−2k1rA0+koffA1−k2rA1+2koffA2,(2.2)dA0dt=−2k1rA0+koffA1,(2.3)dA1dt=2k1rA0−koffA1−k2rA1+2koffA2,(2.4)dA2dt=k2rA1−2koffA2.

The factor of 2 that appears in the reaction terms $2k_{1}rA_{0}$ and $2k^{\text{off}}A_{2}$ is due to our modelling assumption that both antibody arms are identical and their dynamics are independent. As a result, the factor of 2 represents when two antibody arms can undertake a reaction (e.g. an antibody is able to bind either of its arms when in solution and, similarly, dissociate either of them when it is bivalently bound). We close equations (2.1)–(2.4) by imposing the following initial conditions:


(2.5) $$r(0)=r_{tot},\;A_{0}(0)=A_{tot},\;A_{1}(0)=0,\;A_{2}(0)=0.$$

In equation (2.5), we assume that all antigens are initially unbound and we denote by $A_{\text{tot}}$ and $r_{\text{tot}}$ the total number of antibodies and target antigens, respectively, within the system. The parameters $A_{\text{tot}}$ and $r_{\text{tot}}$ have units of antibody number and antigen number per cell, respectively.

By taking suitable linear combinations of equations (2.1)–(2.4), and exploiting equation (2.5), it is straightforward to deduce that the total number of antibodies and antigens are each conserved within the system:


(2.6)A0+A1+A2=Atot,(2.7)r+A1+2A2=rtot.

We use equations (2.6) and (2.7) to eliminate $A_{0}=A_{\text{tot}}-A_{1}-A_{2}$ and $r=r_{\text{tot}}-A_{1}-2A_{2}$ and, henceforth, focus on the following reduced system for $A_{1}(t)$ and $A_{2}(t)$:


dA1dt=2k1(rtot−A1−2A2)(Atot−A1−A2)−koffA1(2.8)−k2(rtot−A1−2A2)A1+2koffA2,(2.9)dA2dt=k2(rtot−A1−2A2)A1−2koffA2,

with the following initial conditions:


(2.10) $$\begin{array}{lcr} & A_{1}(0)=A_{2}(0)=0. & \end{array}$$

Before analysing our reduced model, we pause to estimate the model parameters.

### 2.1. Model parameter estimates

In practice, we model an assay within a well of volume $V_{\text{well}}$ (units: litres, l). Thus, we estimate $A_{\text{tot}}$, the number of antibodies within the system (with units in numbers of ligand or protein), for a given experimental antibody concentration, by noting that


(2.11) $$\begin{array}{lcr} & A_{\text{tot}}=A_{\text{init}}\sigma , & \end{array}$$

where $A_{\text{init}}$ is the initial antibody concentration (units: molar concentration M = mol l^−1^), and σ a ‘mAb molecules per cell’ conversion factor given by


(2.12) $$\begin{array}{lcr} & \sigma =\frac{V_{\text{well}}Na}{T^{0}}, & \end{array}$$

where $Na=6.02214\times 10^{23}$ is Avogadro’s number (units: ${\text{mol}}^{-1}$) and $T^{0}$ is the target cell number within the assay volume. Equation (2.12) is normalized with respect to $T^{0}$, because we are focusing on binding to a single target cell.

Within the literature, estimates of $k^{\text{on}}$ for mAbs typically are stated with units s${,}^{-1}$M${,}^{-1}$. Here, we consider antibody and antigen numbers rather than concentrations. Therefore, we rescale $k^{\text{on}}$ so its units are consistent with those used in our model:


(2.13) $$\begin{array}{lcr} & k_{1}=\frac{k^{\text{on}}}{\sigma }, & \end{array}$$

where the units of $k_{1}$ are the number of antibodies per second.

Two approaches have been used to model the rate at which the second arm of the antibody binds to cell surface antigens (see reaction 2 in figure 2). Sengers *et al*. [18] assume that the second binding event is limited by antigen diffusion on the cell surface and hence, the reaction rate depends on the diffusion of target antigens. The case of antigens diffusing and binding with antibodies on the surface of a cell is an example of a first-hitting time problem that predicts the time taken for a diffusing body (e.g. an antigen) to encounter a trap (here, an antibody’s binding arm). Following Coombs *et al*. [22], the diffusion limited reaction rate for the binding of the second arm, $k_{2}$, can be estimated to be


(2.14) $$\begin{array}{lcr} & k_{2}\approx \frac{D}{4\pi (T_{\text{rad}}{)}^{2}}, & \end{array}$$

where D is the diffusion coefficient of the target antigen (units: ${\text{m}}^{2}\;{\text{s}}^{-1}$) and $T_{\text{rad}}$ is the radius of the target cell (units: m).

Alternatively, Rhoden *et al*. [17] assume the antigens are immobile and, hence, that the reaction rate is limited by the concentration of free antigens within reach of the bound arm of the antibody. In this case, $k_{2}$, the rate of binding of the second arm, is estimated to be


(2.15) $$\begin{array}{lcr} & k_{2}=\frac{k^{\text{on}}[Ag^{\text{eff}}]}{r_{\text{tot}}}=\left(\frac{3}{8\times 10^{3}}\right)\cdot (\frac{k^{\text{on}}}{\pi Na(T_{\text{rad}}{)}^{2}r_{\text{Ab}}}), & \end{array}$$

where $k^{\text{on}}$ was introduced above, $\left[Ag^{\text{eff}}\right]$ is the concentration of free antigen within reach of the bound arm of the antibody and $r_{\text{Ab}}$ is the arm-to-arm binding distance of the antibody. (We refer the interested reader to appendix A for more details about the derivation of equations (2.14) and (2.15).)

From here on, we follow Sengers *et al*. [18] and assume that the second binding event is driven by antigen diffusion on the cell surface. We justify this choice as follows:

— In equation (2.14), the parameter that most commonly varies between target antigens or antibodies is the diffusion coefficient, D only. The corresponding parameters in equation (2.15) are $k^{\text{on}}$ and $r_{\text{Ab}}$. Since $k^{\text{on}}$ is proportional to the monovalent reaction rate, $k_{1}$ (see equation (2.13)), the only parameter related to antibody–target interactions that can alter the ratio of $k_{1}$ to $k_{2}$ is $r_{\text{Ab}}$. In what follows, we will analyse model outputs as the magnitudes of monovalent and bivalent reactions rates vary. In this vein, the antigen diffusion coefficient, D, typically ranges between 10^−15^ and 10^−13^m${,}^{2}$ s${,}^{-1}$ [23] but $r_{\text{Ab}}$, the antibody arm-to-arm distance for an IgG antibody, ranges between $12\;\text{and}\;13\times 10^{-9}$ m [24]. Therefore, by varying D, we explore a wider range of values of the bivalent reaction rate $k_{2}$.
— In Rhoden *et al*. [17], antigens are assumed to be within reach of the antibody binding arm. Assuming that antigens are uniformly distributed over the cell surface, the area swept out by an antibody with $r_{\text{Ab}}=12.5\times 10^{-9}$ m, as a fraction of the surface area of a tumour cell with an average radius of $8\times 10^{-6}$ m [25] is approximately $6\times 10^{-7}$. Therefore, we estimate that approximately $6\times 10^{7}$ antigens per cell would be needed for one antigen to be within reach of an antibody binding arm. This estimate is much larger than the average number of target antigens on the surface of a tumour cell ($10^{4}\;-\;10^{6}$) [26]. We, therefore, argue that the rate limiting step for the surface bound reaction is the ability of the target antigen to diffuse until it is within reach of the antibody binding arm, as described by Sengers *et al*. [18].

To summarize, we extract values for parameters such as $k^{\text{on}}$, $k^{\text{off}}$, D and $A_{\text{init}}$ from the literature and use these to obtain values of $k_{1}$, $k_{2}$ and $A_{\text{tot}}$ for use in our model. For reference, the model parameters and their interpretations are provided in table 1.

**Table 1.** Model parameters associated with equations (2.1)–(2.4). Parameters are from standard human cancer cell lines such as SK-OV3 and mAbs targeting cancer such as trastuzumab (references are given in the table).

parameter	definition	estimated value (units)	source
$r_{\text{tot}}$	target cell antigen density	$10^{4}\;-\;10^{6}$ (antigens)	[26]
$k^{\text{on}}$	antibody in solution binding rate	$10^{4}\;-\;10^{6}$ (s ${,}^{-1}$ M ${,}^{-1}$ )	[10,27]
$k^{\text{off}}$	antibody dissociation rate	$10^{-6}\;-\;10^{-3}$ (s ${,}^{-1}$ )	[10,27]
$A_{\text{init}}$	initial antibody concentration	$10^{-12}\;-\;10^{-5}$ (M)	[28]
$T^{0}$	target cell number in assay	$2\times 10^{5}$ (cells)	[29]
$V_{\text{well}}$	assay reaction well volume	150 (μl)	[29]
$T_{\text{rad}}$	tumour cell radius	8 (μm)	[30]
D	target antigen membrane diffusion coefficient	$10^{-15}\;-\;10^{-13}$ (m ${,}^{2}$ s ${,}^{-1}$ )	[23]
$k_{2}$	diffusion limited second arm binding rate	$10^{-6}\;-\;10^{-4}$ (s ${,}^{-1}$ )	[18]
σ	mAb molecules per cell conversion factor	$4.5\times 10^{14}$ (M ${,}^{-1}$ cell ${,}^{-1}$ )	equation (2.12)

### 2.2. Equilibrium solutions

We now derive equilibrium solutions of equations (2.8) and (2.9). We focus on equilibrium solutions because, for most antibody concentrations, the transient time scale of antibody binding is much shorter (typically less than a second) than the time scales on which effector functions occur (typically minutes to hours) [31,32]. As such, the values of the quantities relating to antibody–antigen binding (e.g. antigen occupancy and the number of bound antibodies) will quickly reach a steady state and we neglect binding dynamics here. We note that for very low antibody concentrations ($10^{-12}$ M), it would take days for a binding reaction to reach equilibrium. The resulting error due to assuming equilibrium in this case, though, is negligible and does not affect the results of this work.

The steady state solutions are determined by setting time derivatives equal to zero in equations (2.8) and (2.9). We denote steady state solutions $A_{1}$ and $A_{2}$ by $A_{1}^{*}$ and $A_{2}^{*}$, respectively, and define ${\hat{A}}_{1}=K_{2}A_{1}^{*}$, ${\hat{A}}_{2}=K_{2}A_{2}^{*}$, ${\hat{r}}_{\text{tot}}=K_{2}r_{\text{tot}}$ and ${\hat{A}}_{\text{init}}=K_{2}A_{\text{init}}\sigma$, where $K_{1}=k_{1}/k^{\text{off}}$ and $K_{2}=k_{2}/k^{\text{off}}$. Exploiting these identities and setting $d\hat{A_{2}}/dt=0$ in equation (2.9), we have that


(2.16) $$\begin{array}{lcr} & {\hat{A}}_{2}=\frac{{\hat{A}}_{1}({\hat{r}}_{\text{tot}}-{\hat{A}}_{1})}{2(1+{\hat{A}}_{1})}. & \end{array}$$

Substituting for $\hat{A_{2}}$ from equation (2.16) into equation (2.8), $d\hat{A_{1}}/dt=0$, upon simplification, we obtain the following cubic polynomial for $\hat{A_{1}}$:


(2.17) $$\begin{array}{lcr} & {\hat{A}}_{1}^{3}+2(1-\frac{{\hat{A}}_{\text{init}}}{1-K_{21}}){\hat{A}}_{1}^{2}+\alpha {\hat{A}}_{1}+\frac{2{\hat{A}}_{\text{init}}}{1-K_{21}}{\hat{r}}_{\text{tot}}=0, & \end{array}$$

where


(2.18) $$\begin{array}{lcr} & \alpha =\frac{2{\hat{A}}_{\text{init}}}{1-K_{21}}({\hat{r}}_{\text{tot}}-1)+1-\frac{({\hat{r}}_{\text{tot}}+1{)}^{2}}{1-K_{21}}. & \end{array}$$

Here, we have introduced $K_{21}=K_{2}/K_{1}$ and substituted $A_{\text{tot}}=A_{\text{init}}\sigma$ from equation (2.11) because in what follows we vary the antibody concentration, $A_{\text{init}}$. It remains to identify the number of real positive roots of equation (2.17). From Descartes’ rule of signs, if there is exactly one sign change between the coefficients of a polynomial (where the coefficients follow the order of the power of the polynomial variable), then that polynomial has exactly one real positive root [33]. Noting that for the parameter values given in table 1, $K_{21}=k_{2}\sigma /k_{\text{on}}\gg 1$, it is possible to show equation (2.17) has exactly one real positive root. As $d{\hat{A}}_{1}/dt>0$ for $A_{1}=0$ in equation (2.8), the trivial solution is unstable and the one real positive root of equation (2.17) is the long time asymptote of equation (2.8). In subsequent sections, we numerically solve equation (2.17), using SciPy’s *fsolve* function to obtain the positive root of equation (2.17) [34]. To the best of our knowledge, equations (2.16) and (2.17) are the first examples within the literature of analytic expressions for the equilibrium binding states of a bivalent ligand without assuming the ligand to be in excess.

In subsequent sections, we are particularly interested in the following quantities:


(2.19)A¯1:=A1∗/(A1∗+A2∗),(2.20)A¯2:=A2∗/(A1∗+A2∗),(2.21)R¯:=(A1∗+2A2∗)/rtot,(2.22)B¯:=(A1∗+A2∗)/rtot,and(2.23)B¯tot:=A1∗+A2∗,

where ${\bar{A}}_{1}$ and ${\bar{A}}_{2}$ are the monovalently and bivalently bound fractions, respectively, $\bar{R}$ is antigen occupancy, $\bar{B}$ is the bound antibody to antigen ratio and ${\bar{B}}_{\text{tot}}$ is the total bound antibody number. We are particularly interested in antigen occupancy and bound antibody numbers because they directly impact the effector function of mAbs. For example, the ratio of bound antibody to antigen has been shown to correlate with effector function potency and efficacy [10].

### 2.3. Global sensitivity analysis method: Sobol sensitivity indices

In this section, we briefly describe Sobol’s method for global parameter sensitivity analysis, prior to its use in §3.2 [35]. Sobol’s method decomposes the variance from a scalar model output into sensitivity indices that quantify the contributions to the output variance of different model parameters. Using this method, we can establish which changes in antibody–antigen interactions have the biggest impact in our quantities of interest (e.g. antigen occupancy).

Sobol’s method utilizes a quasi-random sequence rather than a uniformly distributed sequence of random numbers to improve convergence of the Sobol sensitivity indices (see [36] and [35]).

Given a vector of model inputs, $X=\left\{X_{1},X_{2},...,X_{n}\right\}$*,* any model output can be viewed as a function Y=f(X). The variance of the model output, Var(Y), can be decomposed as follows:


(2.24) $$\begin{array}{lcr} & \text{Var}(Y)=\sum_{i=1}^{n}(V_{i}+\sum_{i<j}^{n}V_{ij}+\ldots ), & \end{array}$$

where


(2.25)Vi=VarXi⁡(EX∼i(Y∣Xi)),(2.26)Vij=VarXij⁡(EX∼ij(Y∣Xi,Xj))−Vi−Vj,i<j

with analogous formulae for higher order terms. Here, $X_{∼i}$ is the set of all variables except $X_{i}$ and $X_{∼ij}$ excludes both $X_{i}$ and $X_{j}$. $E_{X_{∼i}}(Y|X_{i}))$ is the conditional expectation defined as


(2.27) $$E_{X_{∼i}}(Y|X_{i})=\sum_{y\in X_{∼i}}y\frac{P(\{Y=y\}\cap X)}{P(X)},$$

where P is the probability distribution over the model output.

Equation (2.24) details how the total variance in model output can be decomposed into variances in the model output given changes in one parameter $X_{i}$, with $V_{i}$, and $V_{ij}$ denoting the variances due to changes in both $X_{i},X_{j}$ with iteration to changes in three or more parameters.

A direct measure of the sensitivity of the model output to a single parameter, $X_{i}$, is called the ‘first-order sensitivity index’ or ‘main effect index’ $S_{i}$, where


(2.28) $$\begin{array}{lcr} & S_{i}=\frac{V_{i}}{\text{Var}(Y)}. & \end{array}$$

This index measures the effect on the model output Y of varying parameter, $X_{i}$, alone, averaged over variation in all other input parameters. Second-order indices $S_{ij}$ can be defined in a similar way:


(2.29) $$\begin{array}{lcr} & S_{ij}=\frac{V_{ij}}{\text{Var}(Y)}, & \end{array}$$

with analogous expressions for higher order interactions. With $S_{i}$, $S_{ij}$ and higher order terms defined by equations (2.28) and (2.29), equation (2.24) supplies


(2.30) $$\begin{array}{lcr} & 1=\sum_{i=1}^{n}(S_{i}+\sum_{i<j}^{n}S_{ij}+\ldots ). & \end{array}$$

In particular, the sum of first-order and higher order indices sum to 1. The indices detailed above describe how the variance in model output depends on a specific parameter. The ‘total-effect index’ or ‘total order index’, $S_{Ti}$, groups together first- and higher order indices to measure how the parameter $X_{i}$ and its interactions with all other parameters affect the model output. The total order index, $S_{Ti}$, is given by


(2.31) $$\begin{array}{lcr} & S_{Ti}=\frac{E_{X_{∼i}}({\text{Var}}_{X_{i}}(Y|X_{∼i}))}{\text{Var}(Y)}=1-\frac{{\text{Var}}_{X_{∼i}}(E_{X_{i}}(Y|X_{∼i}))}{\text{Var}(Y)}. & \end{array}$$

Unlike first- and higher order sensitivity indices, the sum over all total order indices is larger than or equal to 1. This is because interactions between parameters are accounted for in each parameter's total order indices (i.e. the interaction between $X_{i}$ and $X_{j}$ is accounted for in both $S_{Ti}$ and $S_{Tj}$), so some terms are counted more than once.

To calculate the Sobol indices, we use the following procedure:

(1) Fix upper and lower bounds for the model parameters of interest.
(2) Generate multiple parameter sets by sampling from these parameter ranges using the quasi-random sequence defined in [36,35].
(3) For each parameter set generated at step two, calculate the model outputs of interest.
(4) For each model output generated at step three, calculate the first- and total order sensitivity indices using equations (2.28) and (2.31).

We use the Python package SALib to calculate the sensitivity indices [37,38]. We also include a dummy variable in our analysis [39]. The dummy variable does not appear in the model and is used as a threshold to identify significant parameters above artefact in the sensitivity analysis. In particular, the model’s outputs should not be sensitive to the dummy variable.

## 3. Results

### 3.1. Maximizing the antibody concentration to target antigen ratio increases monovalent binding and bound antibody number

In this section, we analyse the equilibrium solutions predicted by the model to establish which parameters influence the quantities defined by equations (2.19)–(2.22) in different experimental systems, noting that antigen expression levels and antibody numbers can vary over many orders of magnitude. Of interest is how the quantities in equations (2.19)–(2.22) change as $A_{\text{init}}$ and $r_{\text{tot}}$ vary. We will investigate the total bound antibody ${\bar{B}}_{\text{tot}}$ in §3.2.

**Figure 3.**
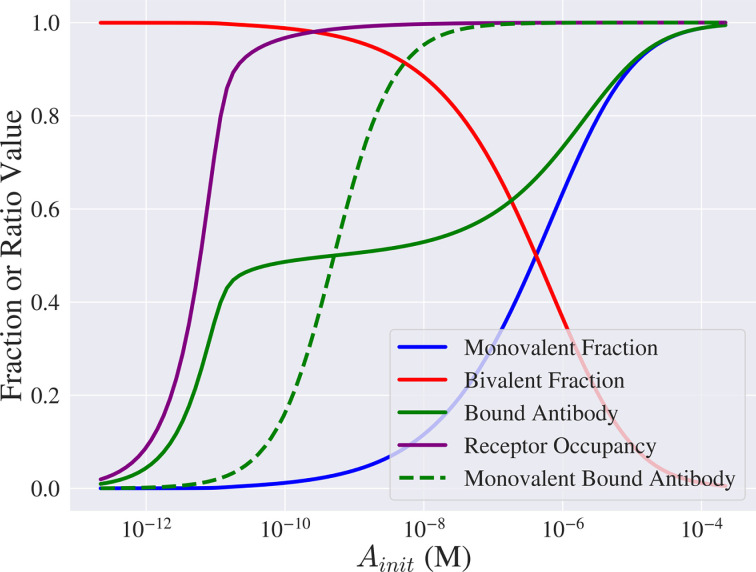
The binding configuration of the antibody is concentration dependent. Series of curves showing how the equilibrium values of fraction of monovalently bound antibodies ($\bar{A_{1}}=A_{1}^{*}/(A_{1}^{*}+A_{2}^{*})$, blue curve), fraction of bivalently bound antibodies ($\bar{A_{2}}=A_{2}^{*}/(A_{1}^{*}+A_{2}^{*})$, red curve), antigen occupancy ($\bar{R}=(A_{1}^{*}+2A_{2}^{*})/r_{\text{tot}}$, purple curve) and the bound antibody to total antigen number ratio ($\bar{B}=(A_{1}^{*}+A_{2}^{*})/r_{\text{tot}}$, green curve) change as $A_{\text{init}}$ varies. For comparison, $\bar{B}$ is plotted for the case of a monovalent antibody that is modelled by setting $k_{2}=0$ and $A_{2}(0)=0$ (green dashed curve). Values of $A_{1}^{*}$ and $A_{2}^{*}$ were obtained by solving equations (2.16) and (2.17) with $k^{\text{on}}=10^{5}$ s${,}^{-1}$M${,}^{-1}$, $k^{\text{off}}=10^{-4}$ s${,}^{-1}$, $r_{\text{tot}}=10^{4}$ and $D=10^{-14}$ m${,}^{2}$ s${,}^{-1}$.

In figure 3, we show how the equilibrium solutions of equations (2.16) and (2.17) and related quantities of interest change as $A_{\text{init}}$ varies for a fixed $r_{\text{tot}}=10^{4}$. Regarding the fractions of monovalently and bivalently bound antibodies, $\bar{A_{1}}$ and $\bar{A_{2}}$, figure 3 shows that when the value of $A_{\text{init}}$ is low ($10^{-12}$ M$-10^{-10}$ M), most antibodies are bivalently bound and there are few monovalently bound antibodies. As the value of $A_{\text{init}}$ increases ($10^{-9}$ M$-10^{-4}$ M), antibodies switch from being bivalently to monovalently bound.

Figure 3 shows further that $\bar{R}$ approaches one (i.e. antigens are saturated) for small values of $A_{\text{init}}$ ($A_{\text{init}}\approx 10^{-10}$ M). This is because antibodies each bind two antigens so few antibodies are required to saturate the target antigens. From figure 3, we also see that antigens remain saturated as antibodies transition from being bivalently to monovalently bound, while the ratio of bound antibody to total antigen level, $\bar{B}$, changes. In particular, $\bar{B}$ reaches a plateau when $A_{\text{init}}\approx 10^{-10}$ M, at which antibodies are primarily bivalently bound, and then increases as the number of monovalently bound antibodies increases. For comparison, in figure 3 we also plot $\bar{B}$ for a monovalent antibody (green dashed line). As expected, monovalent antibodies achieve maximum numbers of bound antibody at a lower value of $A_{\text{init}}$ than bivalent antibodies. This is consistent with the observations of Mazor *et al*. [10] that monovalent antibodies increase the number of antibodies bound to the cell surface and elicit enhanced ADCC potency. A much larger value of $A_{\text{init}}$ ($A_{\text{init}}\approx 10^{-4}$ M) is required for a bivalent antibody to achieve the same number of bound antibodies as a monovalent antibody.

**Figure 4.**
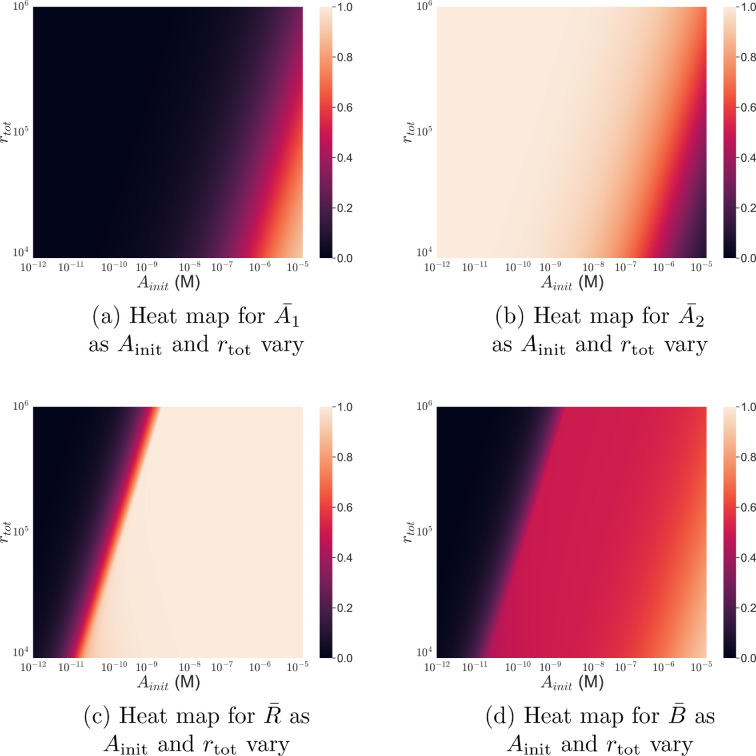
Maximal bound antibody number is achieved for a large ratio of antibody concentration to target density. Heat maps showing how (a) the equilibrium fraction of monovalently bound antibodies $\bar{A_{1}}=A_{1}/(A_{1}+A_{2}$), (b) fraction of bivalently bound antibodies $\bar{A_{2}}=A_{2}/(A_{1}+A_{2}$), (c) antigen occupancy, $\bar{R}=(A_{1}+2A_{2})/r_{\text{tot}}$ and (d) bound antibody to total antigen number ratio $\bar{B}=(A_{1}+A_{2})/r_{\text{tot}}$ change as the parameters $A_{\text{init}}$ and $r_{\text{tot}}$ vary. Values of $A_{1}$ and $A_{2}$ were obtained from equations (2.16) and (2.17) with $k^{\text{on}}=10^{5}$ s${,}^{-1}$M${,}^{-1}$, $k^{\text{off}}=10^{-4}$ s${,}^{-1}$ and $D=10^{-14}$ m${,}^{2}$ s${,}^{-1}$.

In figure 4, we calculate $\bar{A_{1}},\bar{A_{2}},\bar{R}$ and $\bar{B}$ as both $A_{\text{init}}$ and $r_{\text{tot}}$ vary. Figure 4a,b show that there are distinct favourable regimes for the monovalently and bivalently bound fractions, $\bar{A_{1}}$ and $\bar{A_{2}}$; $\bar{A_{1}}$ attains its highest value when the value of $r_{\text{tot}}$ is low and $A_{\text{init}}$ is high. Furthermore, there are more bivalently than monovalently bound antibodies ($\bar{A_{2}}>\bar{A_{1}}$) for most values in the ($r_{\text{tot}}$, $A_{\text{init}}$) plane while the antibody concentration that maximizes $\bar{A_{1}}$ is inversely proportional to the value of $r_{\text{tot}}$. Even for the smallest values of $r_{\text{tot}}$, however, a large value of $A_{\text{init}}$ is required to maximize $\bar{A_{1}}$.

The results in figures 3 and 4a,b are consistent with the experimental work of Bondza *et al*. [12]. Our results predict that the bivalent antibodies we consider are primarily bivalently bound for most antibody concentrations and monovalently bound only for very high concentrations. Similarly, Bondza *et al*. [12] suggested that the binding state of rituximab and obinutuzumab changed in a dose-dependent manner, with the antibody primarily bivalently bound for most antibody concentrations and monovalently bound only for very high concentrations. The results in figure 4a,b extend those of Bondza *et al*. [12] by predicting that the dose-dependent binding behaviour they observed does not depend on the antibody concentration alone. Instead, this behaviour depends on the ratio of total antibody to antigen number within the system with antibodies becoming primarily monovalently bound when this value of ratio is high (large $A_{\text{tot}}$, small $r_{\text{tot}}$). In particular, antibodies may be primarily bivalently bound even for high antibody concentrations if the ratio of total antibody to antigen number is not large enough. This can be seen in figure 4a,b when the value of $r_{\text{tot}}$ is high ($r_{\text{tot}}=10^{6}$).

To assess how the behaviours observed in figure 4a,b translate to quantities that relate to effector function potency and efficacy, in figure 4c,d we plot equilibrium values of antigen occupancy, $\bar{R}$, and the ratio of bound antibody to total antigen number, $\bar{B}$. There is a curve in the ($r_{\text{tot}}$, $A_{\text{init}}$) plane that separates regions in which antigens are saturated ($\bar{R}=1$) from those where antigens are unsaturated ($\bar{R}<1$). In fact, the ratio of total antibody to antigen number is approximately 1/2 on this curve as this corresponds to the minimum number of antibodies required to saturate the target antigens (the value is 1/2 because each antibody can bind two antigens). Therefore, for smaller values of $r_{\text{tot}}$, a smaller value of $A_{\text{init}}$ is required to give 1/2. This is to be expected as smaller numbers of target antigens require fewer antibodies to saturate them. This corresponds to a ‘tight binding regime’, where the binding affinity is larger than the available antigen concentration and, as a result, the reaction is limited by the small number of antigens [40].

As in figure 3, figure 4d shows that $\bar{B}$ attains its maximum value at the same values of $r_{\text{tot}}$ and $A_{\text{init}}$ as $\bar{A_{1}}$. Naturally, the ratio of bound antibody to total antigen number increases with the number of monovalently bound antibodies because more antibodies can bind to the cell surface if they are only binding a single target antigen. Mazor *et al*. [10] showed how binding affinity can be adjusted to maximize the number of bound antibodies (see figure 1). Our analysis extends the results of Mazor *et al*. [10] by predicting which total antigen numbers and antibody concentrations increase the number of monovalently bound antibodies (and as a result the number of bound antibodies), leading to increased effector function potency and efficacy. In particular, our model predicts the largest improvement in effector function when the ratio of total antibody to antigen number is high (e.g. large value of $A_{\text{init}}$ and low value of $r_{\text{tot}}$).

### 3.2. Global sensitivity analysis for antigen occupancy and number of bound antibodies suggests concentration-dependent sensitivities

In this section, we conduct a global parameter sensitivity analysis [41] for a range of antibody concentrations to examine the dependence of key model outputs on model parameters (details of how we calculate the sensitivity indices are included in §2.3) [35]. The outputs of interest are the equilibrium values of $\bar{R}$, $\bar{B}$ and ${\bar{B}}_{\text{tot}}$ (see equations (2.21)–(2.23)).

As before, we focus on these quantities as they have been shown to correlate with the potency and efficacy of mAb treatments [10]. We examine the sensitivity of $\bar{R}$, $\bar{B}$ and ${\bar{B}}_{\text{tot}}$ to variations in $k^{\text{on}}$, $k^{\text{off}}$, $r_{\text{tot}}$ and D. We choose these parameters because $k^{\text{on}}$ and $k^{\text{off}}$ are commonly reported for mAbs while $r_{\text{tot}}$ and D can vary depending on the target antigen. When calculating the sensitivity indices, we vary $k^{\text{on}}$, $k^{\text{off}}$, $r_{\text{tot}}$ and D over the ranges in table 1.

**Figure 5.**
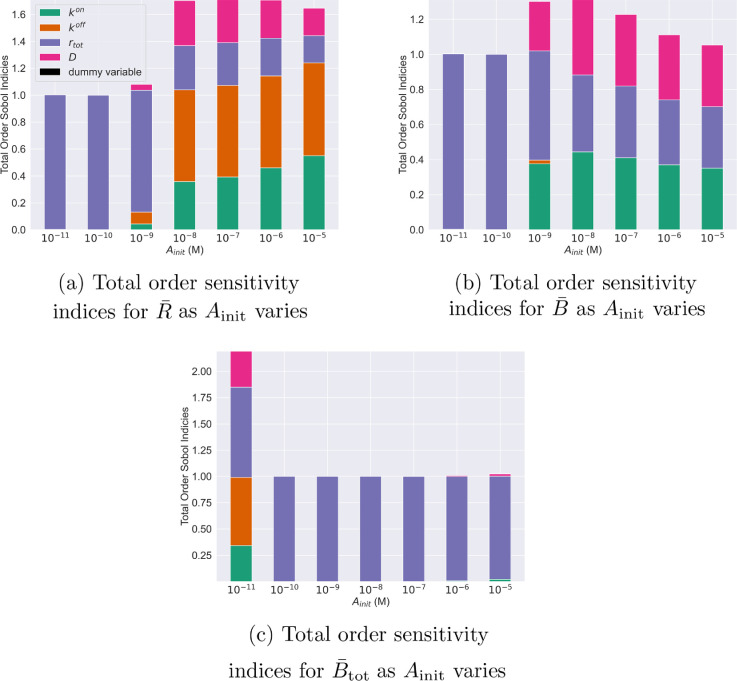
Parameter sensitivities of antigen occupancy and bound antibody to total antigen number ratio are dose-dependent. Total order Sobol sensitivity analysis at different fixed values of $A_{\text{init}}$ for (a) antigen occupancy ($\bar{R}=(A_{1}+2A_{2})/r_{\text{tot}}$), (b) bound antibody to total antigen number ratio ($\bar{B}=(A_{1}+A_{2})/r_{\text{tot}}$) and (c) total number of bound antibodies (${\bar{B}}_{\text{tot}}=A_{1}+A_{2}$). A dummy parameter was added to the analysis to estimate uncertainty within the sensitivity indices as in Marino *et al*. [39].

In figure 5, we present total order sensitivity indices for $\bar{R}$, $\bar{B}$ and ${\bar{B}}_{\text{tot}}$. We calculate these indices for a series of fixed values of the antibody dose $A_{\text{init}}$. Focusing on antigen occupancy, $\bar{R}$ (figure 5a), we observe two qualitatively different regimes. For small values of $A_{\text{init}}$ ($10^{-11}\;-\;10^{-9}$ M), the sensitivity is dominated by the antigen density, $r_{\text{tot}}$. For larger values of $A_{\text{init}}$ ($10^{-8}$ to $10^{-5}$ M), $\bar{R}$ is more sensitive to the binding and unbinding rates.

We observe a similar dependence of the sensitivities of the ratio of bound antibody to the total antigen number, $\bar{B}$, on the antibody concentration (figure 5b). For small values of $A_{\text{init}}$ ($10^{-11}-10^{-9}$ M) the sensitivity is dominated by the antigen density. As $A_{\text{init}}$ increases, $\bar{B}$ becomes sensitive to the binding parameters. Interestingly, comparing figure 5a,b shows that $\bar{B}$ is less sensitive to the off rate, $k^{\text{off}}$, than $\bar{R}$ is.

Figure 5c shows that the total number of bound antibodies ${\bar{B}}_{\text{tot}}$ is most sensitive to the total antigen number, $r_{\text{tot}}$, for all values of $A_{\text{init}}$ except $A_{\text{init}}=10^{-11}$ M. This behaviour is expected since the maximum number of antibodies that can bind to a cell is bounded by the total number of target binding sites when antibodies are in excess. The reason ${\bar{B}}_{\text{tot}}$ is sensitive to the binding parameters when $A_{\text{init}}=10^{-11}$ M is predicted to be because antigens are in excess and, therefore, the number of bound antibodies is limited by the antibody’s ability to bind antigens rather than to the total antigen number.

### 3.3. Conditions that result in a large avidity effect differ between bound antibody number and target occupancy

In this section, we use the model to investigate the avidity effect for antibody binding. Recall that the avidity effect is described as the apparent increase in binding affinity due to multiple bindings. Antibodies can elicit an avidity effect by binding antigens with more than one of their antigen binding arms.

**Figure 6.**
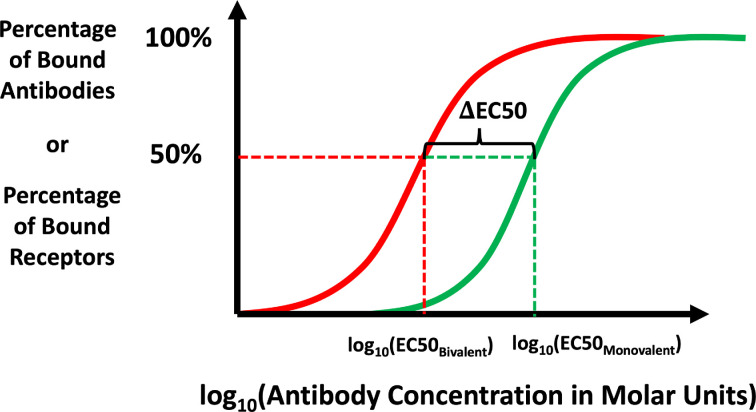
A schematic showing the avidity effect is measured with ΔEC50. The EC50 value is defined as the antibody concentration at which the measurement is half of its maximum value. Here, ${\text{EC50}}_{\text{bivalent}}$ and ${\text{EC50}}_{\text{monovalent}}$ are the iEC50 values for a bivalent and corresponding monovalent antibody (i.e. an antibody with only one functional arm, so that $k_{2}=0,A_{2}=0$ in equations (2.8) and (2.9)). All other parameters are kept the same between the two curves. The shift in EC50, termed ΔEC50, between the monovalent and bivalent case, is due to multiple arms binding antigens, i.e. the avidity effect.


One way to quantify EC50, the binding potency of an antibody, is to measure the antibody concentration at which half of the maximum binding signal is obtained. We measure the avidity effect, ΔEC50, by comparing EC50 values for a monovalent (EC50${\;}_{\text{monovalent}}$) and a bivalent (EC50${\;}_{\text{bivalent}}$) antibody (see figure 6). We define ΔEC50 as

(3.1) $$\begin{array}{lcr} & \Delta \text{EC50}=\log_{10}(\frac{{\text{EC50}}_{\text{Monovalent}}}{{\text{EC50}}_{\text{Bivalent}}}), & \end{array}$$

where ${\text{EC50}}_{\text{bivalent}}$ is calculated using equations (2.8) and (2.9), with ${\text{EC50}}_{\text{monovalent}}$ calculated from the monovalent analogue of these equations, obtained by setting $k_{2}=0$ and $A_{2}(0)=0$. We quantify ΔEC50 in two different ways: by considering the binding signal to be the number of bound antibodies, ${\bar{B}}_{\text{tot}}$, or to be antigen occupancy, $\bar{R}$.

**Figure 7.**
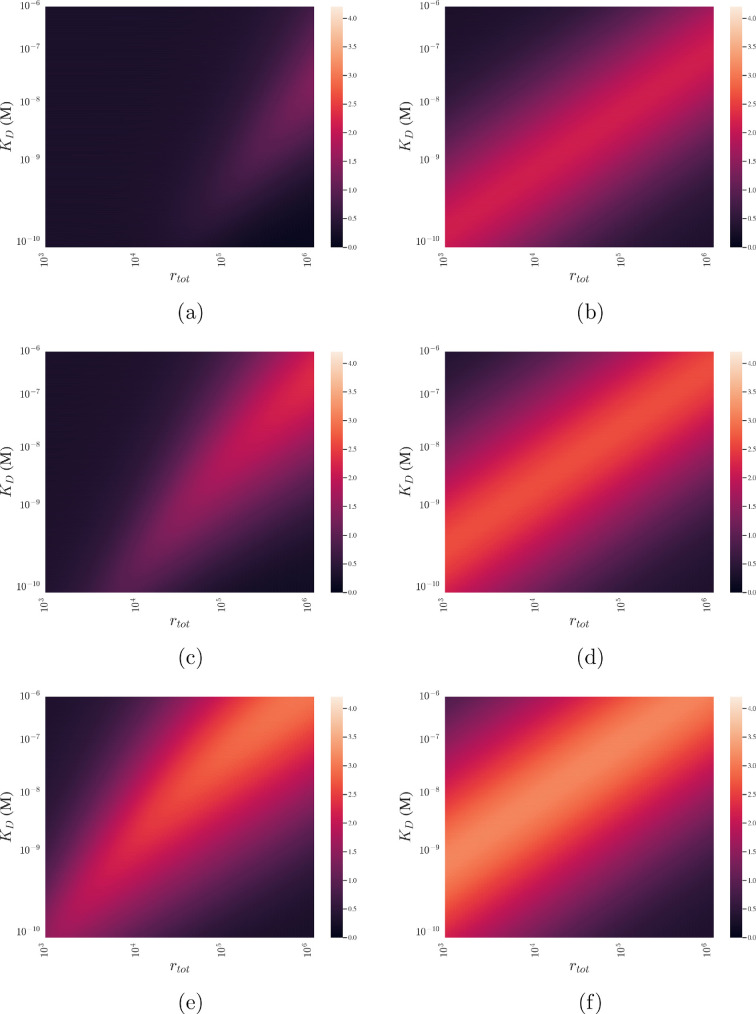
Regions of parameter space that are predicted to induce a large avidity effect differ between antigen occupancy and bound antibody number. Heat maps using equation (3.1) showing how the value of ΔEC50 changes with $r_{\text{tot}}$ and $K_{D}$ for different values of D. ΔEC50 was calculated for the number of bound antibodies (${\bar{B}}_{\text{tot}}=A_{1}+A_{2}$) and ($\bar{R}=(A_{1}+2A_{2})/r_{\text{tot}}$). (a) ΔEC50 for ${\bar{B}}_{\text{tot}}$ with $D=10^{-15}{\text{ m}}^{2}\;{\text{s}}^{-1}$. (b) ΔEC50 for ${\bar{R}}_{\text{tot}}$ with $D=10^{-15}{\text{ m}}^{2}\;{\text{s}}^{-1}$ . (c) ΔEC50 for ${\bar{B}}_{\text{tot}}$ with $D=10^{-14}{\text{ m}}^{2}{\text{s}}^{-1}$. (d) ΔEC50 for ${\bar{R}}_{\text{tot}}$$D=10^{-14}{\text{ m}}^{2}\;{\text{s}}^{-1}$. (e) ΔEC50 for ${\bar{B}}_{\text{tot}}$ with $D=10^{-13}{\text{m}}^{2}\;{\text{s}}^{-1}$. (f) ΔEC50 for ${\bar{R}}_{\text{tot}}$ with $D=10^{-13}{\text{ m}}^{2}\;{\text{s}}^{-1}$.

In figure 7, we show how ΔEC50 changes as we vary the target antigen density, $r_{\text{tot}}$, diffusion coefficient, D, and binding affinity as measured by the dissociation constant, $K_{D}=k^{\text{off}}/k^{\text{on}}$. Focusing on the avidity effect for ${\bar{B}}_{\text{tot}}$, in figure 7a,c,e, there are regions in the ($r_{\text{tot}}$, $K_{D}$) plane, where the value of ΔEC50 is high. In particular, if the antigen density is high and the antibody does not bind strongly ($r_{\text{tot}}=10^{6}$ and $K_{D}=10^{-6}$ M), then the avidity effect is large (ΔEC50=3). In contrast, the avidity effect is smaller when there are few antigens and the antibody binds strongly ($r_{\text{tot}}=10^{3}$ and $K_{D}=10^{-10}$ M) and is smaller again when there are few antigens and the antibody binds weakly ($r_{\text{tot}}=10^{3}$ and $K_{D}=10^{-6}$ M). The region of parameter space in which the avidity effect is large (ΔEC50≥1) increases with D because as the diffusion coefficient increases the second antibody arm binds more readily. As a result, we observe a strong avidity effect in figure 7e compared with figure 7a,c, even when the antibody is a strong binder ($K_{D}=10^{-10}$ M).

Inspection of figure 7b,d,f also reveals that there are values of $r_{\text{tot}}$ and $K_{D}$ for which the avidity effect for $\bar{R}$ is enhanced. With the exception of $D=10^{-13}$ m${,}^{2}$ s${,}^{-1}$, these regions are markedly different to those for ${\bar{B}}_{\text{tot}}$ (figure 7a,c,e). In particular, the avidity effect for $\bar{R}$ is large when the antibody is a strong binder (low value of $K_{D}$) for all reported values of D shown. However, for small values of $K_{D}$, a large avidity effect for ${\bar{B}}_{\text{tot}}$ is only seen when $D=10^{-13}$ m${,}^{2}$ s${,}^{-1}$ (figure 7e).

Figure 7 suggests that a necessary condition for the avidity effect of ${\bar{B}}_{\text{tot}}$ to be large is that bivalent binding is favoured.

## 4. Discussion and conclusions

Monoclonal antibodies are an important class of immunotherapies that are being used to treat cancer. Central to their mechanism of action are their interactions with their target antigens. During the drug discovery process, the binding affinity between the target and antibody is often prioritized. However, other factors, such as the antigen density and valency of the antibody, are known to be important for effector function potency and efficacy [10].

In this article, we have used a mathematical model of a bivalent, monospecific antibody binding to cell membrane antigens given by equations (2.1)–(2.4), to investigate how changing model parameters impacts antigen occupancy, the number of bound antibodies and the avidity effect. We focused on these quantities as they have been shown to relate to the potency and efficacy of antibody effector functions such as ADCC [10]. We used the model to investigate how antibody concentrations and antigen numbers impact antigen occupancy, the ratio of bound antibody to antigen and whether the antibody is monovalently or bivalently bound (figures 3 and 4). We found that higher values of the ratio of total antibody to antigen number within the system resulted in larger numbers of monovalently bound antibodies and, consequently, an increase in the number of antibodies bound to the cell surface. This result suggests that a possible way to increase ADCC efficacy is to ensure that the antibody dose is large enough that monovalent binding is favoured. In turn, increased numbers of monovalently bound antibodies will increase the number of antibody Fc regions available to activate an effector cell to lyse a tumour cell. However, we note that it may be unfeasible to administer a sufficiently large dose to achieve improved efficacy with high levels of monovalent binding due to, for example, increased toxicity. Alternatively, monovalent antibodies can lead to increased levels of monovalent binding at a smaller dose.

Next, we performed a global parameter sensitivity analysis to determine how quantities that relate to mAb potency and efficacy, such as antigen occupancy, change when parameters that govern antibody–target interactions (on and off rates $k^{\text{on}}$ and $k^{\text{off}}$, surface diffusion of antigen D and total antigen number $r_{\text{tot}}$) are varied. We performed the sensitivity analysis for different fixed values of the antibody concentration, $A_{\text{init}}$, to see how the sensitivities changed with antibody dose. We identified two regimes of sensitivity for antigen occupancy: a low antibody concentration regime, where antigens are in excess of antibody and antigen occupancy is most sensitive to antigen density, and a high antibody concentration regime, where antibody is in excess of antigens and antigen occupancy becomes sensitive to the binding parameters. Both regimes may be inferred from figure 5a, where for low values of the antibody concentration, $A_{\text{init}}$ ($A_{\text{init}}\approx 10^{-11}\;-\;10^{-9}$ M), the total order sensitivity index for the total antigen number is large, with a value of approximately one. As the initial antibody concentration increases ($A_{\text{init}}\geq 10^{-8}$ M) the total order sensitivity indices for $k^{\text{on}}$ and $k^{\text{off}}$ become large, attaining values of approximately 0.4 and 0.6, respectively.

The sensitivity analysis for the bound antibody to total antigen number ratio also shows two regimes depending on whether the value of $A_{\text{init}}$, and thus the antibody level, is high or low (figure 5b). Similar to antigen occupancy, the sensitivities to the binding parameters increase at higher antibody concentrations ($A_{\text{init}}\geq 10^{-9}$ M). Interestingly, for higher antibody concentrations, the sensitivity to $k^{\text{off}}$ for the ratio of bound antibody to total antigen number is negligible whereas antigen occupancy is sensitive to $k^{\text{off}}$ at the same antibody concentrations.

The dependence of the sensitivities on antibody concentration has important implications for the development of antibody therapeutics. If the tumour microenvironment, or other factors such as antibody pharmacokinetics, hinder antibody infiltration, then the ratio of antibody to antigen will be low. In such situations, targets of high abundance should be prioritized and increasing the affinity of the antibody may not significantly enhance the mAb’s anti-tumour effects. Conversely, if antibodies are in surplus, then altering the binding affinity is predicted to impact antigen occupancy and the bound antibody to total antigen number ratio, potentially improving mAb potency. This is the case for immune checkpoint inhibitors, that benefit from increased antigen occupancy, and effector functions, such as ADCC, that benefit from maximizing the number of bound antibodies per antigen.

Our analysis predicts that there are (at least) two ways to increase the potency and efficacy of effector functions that relate to the number of bound antibodies. Figure 5b shows that the choice of parameters to be varied in order to maximize potency and efficacy depends on the antibody concentration. In particular, figure 5b indicates a lack of sensitivity to the binding parameters at low antibody concentrations. Therefore, altering the binding affinity to improve effector function potency and efficacy, as implemented by Mazor *et al*. [10], is predicted to have no effect for these concentrations. Only when the antibody concentration is sufficiently large (e.g. $A_{\text{init}}\geq 10^{-8}$ M) will altering the affinity potentially improve effector function. Additionally, figure 5c highlights that increasing the total antigen number will have the greatest effect on the total number of bound antibodies.

The above observations suggest ways to increase the number of effector cell activating antibody Fc regions on the surface of a tumour cell. While increasing the ratio of bound antibody to antigen will maximize the number of antibody Fc regions on the surface of a tumour cell for a given total antigen density, increasing the total antigen number will increase the upper bound for the number of antibody Fc regions. The results presented here suggest that utilizing both strategies will be advantageous for mAb effector functions. In particular, choosing targets that are overly expressed on tumour cells is already common practice and will increase the maximum number of antibody Fc regions that can potentially be presented to the effector cell. Ensuring the antibody to antigen ratio is high and altering the binding affinity to enhance monovalent binding, as in Mazor *et al*. [10], will maximize monovalent binding and ensure that the number of antibody Fc regions on the surface of the tumour cell approaches its maximum given by the total antigen number.

Another focus of this work has been to study the avidity effect seen in antibody binding. We have shown that there exist ranges of the total antigen number $r_{\text{tot}}$ and the binding affinity, as measured with $K_{D}=k^{\text{off}}/k^{\text{on}}$, where the avidity effect is predicted to significantly increase the difference in the EC50 between bivalent and monovalent antibodies for both antigen occupancy and the number of bound antibodies. Figure 7 shows that the avidity effect is predicted to generate a significant change in EC50 for different ranges of target antigen numbers and binding affinities depending on whether the antigen occupancy or the number of bound antibodies is measured. In particular, the size of the avidity effect is small for the number of bound antibodies compared to antigen occupancy when there are few target antigens (low value of $r_{\text{tot}}$) and the antibody is a strong binder (low value of $K_{D}$) and even worse for very high values of $K_{D}$. It follows that, in these situations, an antibody whose anti-tumour effects correlate with the number of bound antibodies is predicted to gain little benefit from the avidity effect. In contrast, an antibody whose potency and efficacy rely on antigen occupancy, such as immune checkpoint inhibitors, should gain an improvement in their anti-tumour effects from the avidity effect at low values of $K_{D}$ and target antigen. These results can be used to aid target selection in preclinical development of therapies that utilize the avidity effect.

A potential limitation of the model is its applicability to certain agonistic antibodies. While our modelling results, along with the findings of Mazor *et al*. [10], suggest that increased bound antibody numbers enhance functional responses in ADCC, this relationship may not be as straightforward for agonistic antibodies like the anti-CD40 mAb selicrelumab [42]. In such cases, the response is likely nonlinear with respect to target occupancy or bound antibody number. Further research is needed to determine the precise functional relationship. Nevertheless, in such cases, our model provides a strong prospective foundation for this future work. Our model, in its current form, is also not as applicable to antigens that form dimers or multi-unit complexes. Additionally, it is important to note that the model’s predicted avidity is an approximation, and the actual avidity effect may be lower than the ideal due to the alignment and rotational adjustments required for paratope–epitope binding on the cell surface. Since this effect is both epitope- and mAb-dependent, the model nonetheless remains adaptable through appropriate calibration of $k_{2}$.

In summary, we have used mathematical modelling to study the interaction of mAbs with their antigens in order to understand how this may regulate mAb effector functions in cancer immunotherapies, noting that factors affecting the number of bound antibodies on a target cell, antigen occupancy and the avidity effect can impact the potency and efficacy of the mAb therapy. With prospective relevance for the preclinical development of immuno-oncological therapies, the main results of this study are threefold. First, we have predicted the importance of the ratio of antibody to antigen within the system for the antibody’s binding state and the values of this ratio that enhance antigen occupancy and bound antibody numbers, providing a quantitative framework to understand and expand previous experimental work by Mazor *et al*. [10] and Bondza *et al*. [12]. In addition, we have identified parameter sensitivities of measures of mAb potency and efficacy and predict that these sensitivities depend on antibody concentration. Finally, we have highlighted regions of parameter space that are predicted to possess a large avidity effect, with parameters associated with large avidity differing between two measures of binding signal, the number of bound antibodies and antigen occupancy.

## Figures and Tables

**Figure 8.**
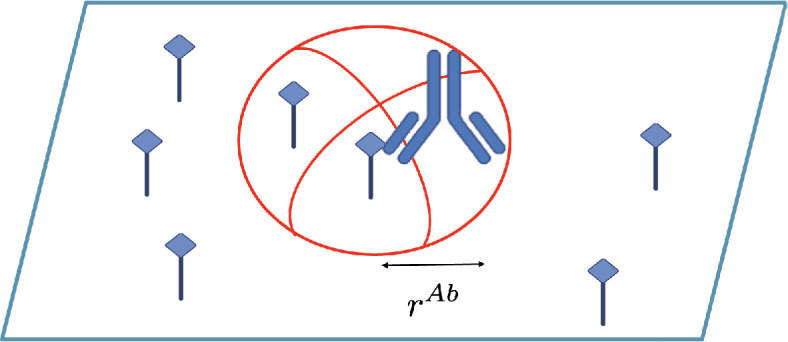
Depiction of the constrained volume that the antibody can sample from after the first binding event. $r_{\text{Ab}}$ denotes the arm-to-arm distance of a standard IgG antibody.

## Data Availability

Code used to generate the figures in this paper is stored on Zenodo [43].

## Appendix A. Modelling methodologies for the second binding event

In this appendix, we look in more detail at modelling the binding of the second arm, as described in Sengers *et al.* [18] and Rhoden *et al.* [17].

### A.1. Method 1: surface diffusion limited binding

In Sengers *et al.* [18], an ODE model is developed to describe bispecific antibody binding to two different targets, CD4 and CD70, on the surface of a target cell. For an antibody bound to a cell with one arm and an additional free antigen to bind, their separation on the cell surface must be on the molecular scale. Sengers *et al.* [18] assume that, due to this small binding distance, the reaction where the antibody binds its second arm with a target antigen on the surface of the cell is independent of the in-solution binding rate. Instead, they suppose that the rate limiting step is reactant diffusion, i.e. the ability of the reactants to diffuse on the cell surface and get within binding distance of each other. In particular, the rate at which the antibody is able to bind a second antigen on the cell surface is assumed to be proportional to the sum of the diffusion coefficients of the reactants.

Sengers *et al.* [18] also estimate that the time scale on which an antigen can freely diffuse across large distances across the cell surface is much smaller than the average half-life of an antibody–antigen complex. As such, a second diffusion-driven binding event is possible even when antigens are sparsely distributed across the cell surface.

For antigens diffusing on the surface of a cell and binding with each other, this is an example of a first-hitting time problem that is extensively studied in Coombs *et al*. [22]. Here, we follow the analysis presented in Coombs *et al*. [22] to derive a reaction rate for the binding of the second arm of the antibody on the cell surface.

Following Coombs *et al*. [22], for a molecule diffusing on the unit sphere, S, with N≫1 number of absorbing traps, the mean first passage time, u, is given by

(A 1)
$$\begin{array}{lcr} & \bar{u}=\frac{2}{D}[\frac{1}{N\mu }+(\log\;2-\frac{1}{2})-\frac{2}{N^{2}}p], & \end{array}$$

where the bar represents the mean taken over the unit sphere. For a trap of radius ϵa (ϵ being a small parameter), μ is defined to be

(A.2)
$$\mu =\frac{1}{\log\left(\frac{1}{\epsilon a}\right)}.$$

Additionally, p is a negative interaction energy given by

(A 3)
$$\begin{array}{lcr} & p=\sum_{i=1}^{N}\sum_{j>i}^{N}\log|x_{i}-x_{j}|. & \end{array}$$

With further details given in Coombs *et al*. [22] and Bergersen *et al*. [44], one can assume that when N≫1, the system relaxes to a thermodynamic ground state where p is maximal such that

(A 4)
$$p\approx \text{max}(p)=\frac{1}{4}\log\left(\frac{4}{e}\right)N^{2}+\frac{1}{4}N\log N+O(N).$$

Substituting equation (A 4) into equation (A 1) and simplifying gives

(A 5)
$$\bar{u}=\frac{2}{D}[\frac{1}{N\mu }-\frac{1}{2N}\log N+O\left(\frac{0.1}{N}\right)].$$

For N≫1, Coombs *et al*. [22] note that equation (A 5) is valid only when ϵ is sufficiently small such that 1/(Nμ) is the dominant term. This condition requires both 1/μ≫1 and $1\gg N(\epsilon a{)}^{2}$ (or in dimensional units $T_{\text{rad}}^{2}\gg N(\epsilon a{)}^{2}$, where $T_{\text{rad}}$ is the cell radius in metres), i.e. the receptor area is small compared with the total cell surface area. Due to the very small surface area occupied by a receptor, the latter is a valid assumption to make. Furthermore, in dimensional units, $1/\mu =\text{log}(T_{\text{rad}}/r_{\text{Ab}})\approx 2\pi \gg 0.1$. At leading order, it follows that

(A 6)
$$\bar{u}\approx \frac{2}{DN}\log\left(\frac{1}{\epsilon a}\right).$$

Restoring dimensions,

(A 7)
$$\bar{u}\approx \frac{2T_{\text{rad}}^{2}}{DN}\log\left(\frac{T_{\text{rad}}}{r_{\text{Ab}}}\right).$$

To relate the mean first passage time to our required reaction rate $k_{2}$, we note from Lamirande *et al*. [45] (electronic supplementary material), that a link between the mean first passage time, $\bar{u}$, and the mean value approximation of a complex that is exponentially decaying with half-life $t_{1/2}$, is

(A 8)
$$\bar{u}=\frac{t_{1/2}}{\ln2}.$$

From equation (2.3), ignoring the dynamics that do not involve the term containing $k_{2}$, for a fixed receptor number, r, we have that

(A 9)
$$A_{1}\approx Ce^{-Kt},$$

where C is a constant and $K=k_{2}r$ (note that here r≡N from [22]). It then follows from equation (A 9) that

(A 10)
$$t_{1/2}=\frac{\ln2}{K}.$$

Finally, substituting equations (A 7) and (A 10) into equation (A 8) and rearranging for $k_{2}$, we arrive at

(A 11)
$$k_{2}\approx \frac{D}{2T_{\text{rad}}^{2}}\frac{1}{\log\left(\frac{T_{\text{rad}}}{r_{\text{Ab}}}\right)},$$

where D is the diffusion coefficient of the target antigen (units: ${\text{m}}^{2}\;{\text{s}}^{-1}$), $T_{\text{rad}}=8\times 10^{-6}$ is the average radius of a tumour cell (units: m) and $r_{\text{Ab}}=1.25\times 10^{-8}$ is the arm-to-arm antibody length (units: m). For the standard values of $T_{\text{rad}}$ and $r_{\text{Ab}}$ listed here, we have that $\log(\frac{T_{\text{rad}}}{r_{\text{Ab}}})\approx 2\pi$ such that

(A 12)
$$\begin{array}{lcr} & k_{2}\approx \frac{D}{4\pi T_{\text{rad}}^{2}}, & \end{array}$$

with $k_{2}$ being the reaction rate for a monovalently bound antibody binding its second antigen binding arm.

### A.2. Method 2: free antigen concentration within the antibody arm reach

Rhoden *et al*. [17] use an ODE model to investigate the binding kinetics of a bispecific antibody as target antigen numbers and binding affinities vary. In their model, the binding of the bispecific antibody’s arms is viewed as an independent event. When modelling the binding of the second arm, Rhoden *et al*. [17] assume that the antibody is fixed to a region near the cell surface and the unbound arm of the antibody is confined to a volume defined by its size, flexibility and structure (see figure 8). In more detail, the antibody samples a hemisphere of radius equal to the length of a typical IgG. The concentration of target antigens within the hemispherical volume is estimated from data recording the average number of antigens per cell.

The effective target concentration, $\left[Ag_{\text{eff}}\right]$, after the first binding event is estimated using the following formula:

(A 13)
$$\begin{array}{lcr} & [Ag_{\text{eff}}]=(\frac{\text{antigen number per cell }\cdot \text{reaction surface area}}{Na\cdot \text{cell surface area}\cdot \text{reaction volume}}), & \end{array}$$

where the reaction surface area, cell surface area and reaction volume are given by $\pi r_{\text{Ab}}^{2}$, $4\pi T_{\text{rad}}^{2}$ and $2\pi r_{\text{Ab}}^{3}/3$, respectively. It follows that

(A 14)
$$\begin{array}{lcr} & [Ag_{\text{eff}}]=\left(\frac{3\times 10^{-3}}{8\pi Na}\right)\times (\frac{r_{\text{tot}}}{(T_{\text{rad}}{)}^{2}r_{\text{Ab}}}). & \end{array}$$

Here, $r_{\text{tot}}$ is the number of target antigens per cell, $T_{\text{rad}}$ and $r_{\text{Ab}}$ are the radius of the target cell and arm-to-arm antibody length, respectively (units: m). The factor $10^{-3}$ converts 1/metres cubed (${\text{m}}^{-3}$) to 1/litres (l^−1^) so $\left[Ag_{\text{eff}}\right]$ has units of molar concentration. The rate constant for the second binding event, $k_{2}$, is then

(A 15)
$$k_{2}=\frac{k_{\text{on}}[Ag_{\text{eff}}]}{r_{\text{tot}}}=\left(\frac{3\times 10^{-3}}{8\pi Na}\right)\times (\frac{k_{\text{on}}}{(T_{\text{rad}}{)}^{2}r_{\text{Ab}}}),$$

where $k^{\text{on}}$ is the in-solution monovalent binding reaction rate (units: ${\text{s}}^{-1}{\text{M}}^{-1}$). In equation (A 15) , we divide by $r_{\text{tot}}$ so that $k_{2}$ is a reaction rate per antibody–antigen complex.

## References

[1] Gonzalez H, Hagerling C, Werb Z. 2018 Roles of the immune system in cancer: from tumor initiation to metastatic progression. Genes Dev. **32**, 1267–1284. (10.1101/gad.314617.118) 30275043 PMC6169832

[2] Chi H, Pepper M, Thomas PG. 2024 Principles and therapeutic applications of adaptive immunity. Cell **187**, 2052–2078. (10.1016/j.cell.2024.03.037) 38670065 PMC11177542

[3] Carpenter S, O’Neill LAJ. 2024 From periphery to center stage: 50 years of advancements in innate immunity. Cell **187**, 2030–2051. (10.1016/j.cell.2024.03.036) 38670064 PMC11060700

[4] Hiam-Galvez KJ, Allen BM, Spitzer MH. 2021 Systemic immunity in cancer. Nat. Rev. Cancer **21**, 345–359. (10.1038/s41568-021-00347-z) 33837297 PMC8034277

[5] Vinay DS *et al*. 2015 Immune evasion in cancer: mechanistic basis and therapeutic strategies. Semin. Cancer Biol. **35**, S185–S198. (10.1016/j.semcancer.2015.03.004) 25818339

[6] Rajewsky K. 2019 The advent and rise of monoclonal antibodies. Nature **575**, 47–49. (10.1038/d41586-019-02840-w) 31686050

[7] Haanen J, Robert C. 2015 Immune checkpoint inhibitors. In Progress in tumor research, pp. 55–66, vol. 42. Basel, Switzerland: Karger. (10.1159/000437178) 26382943

[8] van Erp EA, Luytjes W, Ferwerda G, van Kasteren PB. 2019 Fc-mediated antibody effector functions during respiratory syncytial virus infection and disease. Front. Immunol. **10**, 1664–3224. (10.3389/fimmu.2019.00548) 30967872 PMC6438959

[9] Lo Nigro C, Macagno M, Sangiolo D, Bertolaccini L, Aglietta M, Merlano MC. 2019 NK-mediated antibody-dependent cell-mediated cytotoxicity in solid tumors: biological evidence and clinical perspectives. Ann. Transl. Med. **7**, 105–105. (10.21037/atm.2019.01.42) 31019955 PMC6462666

[10] Mazor Y, Yang C, Borrok MJ, Ayriss J, Aherne K, Wu H, Dall’Acqua WF. 2016 Enhancement of immune effector functions by modulating IgG’s intrinsic affinity for target antigen. PLoS ONE **11**, e0157788. (10.1371/journal.pone.0157788) 27322177 PMC4913924

[11] Junker F *et al*. 2021 A human receptor occupancy assay to measure anti‐PD‐1 binding in patients with prior anti‐PD‐1. Cytom. A **99**, 832–843. (10.1002/cyto.a.24334) PMC8451911 33704890

[12] Bondza S, ten Broeke T, Nestor M, Leusen JHW, Buijs J. 2020 Bivalent binding on cells varies between anti-CD20 antibodies and is dose-dependent. mAbs **12**, 1792673. (10.1080/19420862.2020.1792673) 32744151 PMC7531561

[13] Oostindie SC, Lazar GA, Schuurman J, Parren PWHI. 2022 Avidity in antibody effector functions and biotherapeutic drug design. Nat. Rev. Drug Discov. **21**, 715–735. (10.1038/s41573-022-00501-8) 35790857 PMC9255845

[14] Klein JS, Bjorkman PJ. 2010 Few and far between: how HIV may be evading antibody avidity. PLoS Pathog. **6**, e1000908. (10.1371/journal.ppat.1000908) 20523901 PMC2877745

[15] Perelson AS, DeLisi C. 1980 Receptor clustering on a cell surface. I. Theory of receptor cross-linking by ligands bearing two chemically identical functional groups. Math. Biosci. **48**, 71–110. (10.1016/0025-5564(80)90017-6)

[16] Kaufman EN, Jain RK. 1992 Effect of bivalent interaction upon apparent antibody affinity: experimental confirmation of theory using fluorescence photobleaching and implications for antibody binding assays. Cancer Res. **52**, 4157–4167. 1638531

[17] Rhoden JJ, Dyas GL, Wroblewski VJ. 2016 A modeling and experimental investigation of the effects of antigen density, binding affinity, and antigen expression ratio on bispecific antibody binding to cell surface targets. J. Biol. Chem. **291**, 11337–11347. (10.1074/jbc.m116.714287) 27022022 PMC4900278

[18] Sengers BG, McGinty S, Nouri FZ, Argungu M, Hawkins E, Hadji A, Weber A, Taylor A, Sepp A. 2016 Modeling bispecific monoclonal antibody interaction with two cell membrane targets indicates the importance of surface diffusion. mAbs **8**, 905–915. (10.1080/19420862.2016.1178437) 27097222 PMC4968105

[19] Birtwistle MR, Kholodenko BN. 2009 Endocytosis and signalling: a meeting with mathematics. Mol. Oncol. **3**, 308–320. (10.1016/j.molonc.2009.05.009) 19596615 PMC2745732

[20] Vainshtein I, Roskos LK, Cheng J, Sleeman MA, Wang B, Liang M. 2015 Quantitative measurement of the target-mediated internalization kinetics of biopharmaceuticals. Pharm. Res. **32**, 286–299. (10.1007/s11095-014-1462-8) 25208874 PMC4284384

[21] Hansen SH, Sandvig K, van Deurs B. 1992 Internalization efficiency of the transferrin receptor. Exp. Cell Res. **199**, 19–28. (10.1016/0014-4827(92)90457-j) 1735458

[22] Coombs D, Straube R, Ward M. 2009 Diffusion on a sphere with localized traps: mean first passage time, eigenvalue asymptotics, and Fekete points. SIAM J. Appl. Math. **70**, 302–332. (10.1137/080733280)

[23] McCloskey MA, Poo MM. 1986 Rates of membrane-associated reactions: reduction of dimensionality revisited. J. Cell Biol. **102**, 88–96. (10.1083/jcb.102.1.88) 3001105 PMC2114064

[24] Sosnick TR, Benjamin DC, Novotny J, Seeger PA, Trewhella J. 1992 Distances between the antigen-binding sites of three murine antibody subclasses measured using neutron and X-ray scattering. Biochemistry **31**, 1779–1786. (10.1021/bi00121a028) 1737031

[25] Zhou J, Kulasinghe A, Bogseth A, O’Byrne K, Punyadeera C, Papautsky I. 2019 Isolation of circulating tumor cells in non-small-cell-lung-cancer patients using a multi-flow microfluidic channel. Microsystems Nanoeng. **5**, 8. (10.1038/s41378-019-0045-6) PMC6387977 31057935

[26] Mazor Y *et al*. 2015 Improving target cell specificity using a novel monovalent bispecific IgG design. mAbs **7**, 377–389. (10.1080/19420862.2015.1007816) 25621507 PMC4622537

[27] Bostrom J, Haber L, Koenig P, Kelley RF, Fuh G. 2011 High affinity antigen recognition of the dual specific variants of Herceptin is entropy-driven in spite of structural plasticity. PLoS One **6**, e17887. (10.1371/journal.pone.0017887) 21526167 PMC3081289

[28] Pollard TD. 2010 A guide to simple and informative binding assays. Mol. Biol. Cell **21**, 4061–4067. (10.1091/mbc.e10-08-0683) 21115850 PMC2993736

[29] Yu X, Pegram CN, Bigner DD, Chandramohan V. 2017 Development and validation of a cell-based fluorescent method for measuring antibody affinity. J. Immunol. Methods **442**, 49–53. (10.1016/j.jim.2016.12.004) 28024998 PMC5293658

[30] Hosokawa M *et al*. 2013 Size-based isolation of circulating tumor cells in lung cancer patients using a microcavity array system. PLoS ONE **8**, e67466. (10.1371/journal.pone.0067466) 23840710 PMC3696066

[31] James L, Tawfik D. 2005 Structure and kinetics of a transient antibody binding intermediate reveal a kinetic discrimination mechanism in antigen recognition. Proc. Natl Acad. Sci. USA **102**, 12730–12735. (10.1073/pnas.0500909102) 16129832 PMC1200256

[32] Hoffman F, Gavaghan D, Osborne J, Barrett IP, You T, Ghadially H, Sainson R, Wilkinson RW, Byrne HM. 2018 A mathematical model of antibody-dependent cellular cytotoxicity (ADCC). J. Theor. Biol. **436**, 39–50. (10.1016/j.jtbi.2017.09.031) 28970093

[33] Anderson B, Jackson J, Sitharam M. 1998 Descartes’ rule of signs revisited. Am. Math. Mon. **105**, 447–451. (10.1080/00029890.1998.12004907)

[34] Virtanen P *et al*. 2020 SciPy 1.0: fundamental algorithms for scientific computing in Python. Nat. Methods **17**, 261–272. (10.1038/s41592-019-0686-2) 32015543 PMC7056644

[35] Sobol′ IM. 2001 Global sensitivity indices for nonlinear mathematical models and their Monte Carlo estimates. Math. Comput. Simul. **55**, 271–280. (10.1016/s0378-4754(00)00270-6)

[36] Homma T, Saltelli A. 1996 Importance measures in global sensitivity analysis of nonlinear models. Reliab. Eng. Syst. Saf. **52**, 1–17. (10.1016/0951-8320(96)00002-6)

[37] Herman J, Usher W. 2017 SALib: an open-source Python library for sensitivity analysis. J. Open Source Softw. **2**, 97. (10.21105/joss.00097)

[38] Iwanaga T, Usher W, Herman J. 2022 Toward SALib 2.0: advancing the accessibility and interpretability of global sensitivity analyses. Socio Environ. Syst. Model. **4**, 18155. (10.18174/sesmo.18155)

[39] Marino S, Hogue IB, Ray CJ, Kirschner DE. 2008 A methodology for performing global uncertainty and sensitivity analysis in systems biology. J. Theor. Biol. **254**, 178–196. (10.1016/j.jtbi.2008.04.011) 18572196 PMC2570191

[40] Jarmoskaite I, AlSadhan I, Vaidyanathan PP, Herschlag D. 2020 How to measure and evaluate binding affinities. elife **9**, e57264. (10.7554/elife.57264) 32758356 PMC7452723

[41] Iooss B, Lemaître P. 2015 A review on global sensitivity analysis methods. In Uncertainty management in simulation-optimization of complex systems (eds G Dellino, C Meloni), pp. 101–122. Boston, MA: Springer. (10.1007/978-1-4899-7547-8_5)

[42] Byrne KT *et al*. 2021 Neoadjuvant selicrelumab, an agonist CD40 antibody, induces changes in the tumor microenvironment in patients with resectable pancreatic cancer. Clin. Cancer Res. **27**, 4574–4586. (10.1158/1078-0432.ccr-21-1047) 34112709 PMC8667686

[43] lukedtc. 2025 lukedtc/Antibody_binding_repo: Code for ‘Understanding Antibody-Target Antigen Interactions and the Avidity Effect Using Mathematical Modelling’. (v1.0.0). Zenodo. (10.5281/zenodo.15350514) PMC12308350 40628289

[44] Bergersen B, Boal D, Palffy-Muhoray P. 1994 Equilibrium configurations of particles on a sphere: the case of logarithmic interactions. J. Phys. **27**, 2579–2586. (10.1088/0305-4470/27/7/032)

[45] Lamirande P, Eamonn AG, Gertz M, Philip KM, Jessica RC, Antonello C. 2024 A first passage model of intravitreal drug delivery and residence time, in relation to ocular geometry, individual variability, and injection location. (10.48550/arXiv.2404.04086) PMC11488524 39412819

